# Validation and reproducibility of a semi-qualitative food frequency questionnaire for assessment of sodium intake in Iranian population

**DOI:** 10.1186/s12937-021-00749-7

**Published:** 2022-02-04

**Authors:** Noushin Mohammadifard, Narges Grau, Alireza Khosravi, Ahmad Esmaillzadeh, Awat Feizi, Zahra Abdollahi, Nizal Sarrafzadegan

**Affiliations:** 1 Hypertension Research Center, Cardiovascular Research Institute, Isfahan University of Medical Sciences, Isfahan, Iran; 2 Isfahan Cardiovascular Research Center, Cardiovascular Research Institute, Isfahan University of Medical Sciences, Isfahan, Iran; 3 Interventional Cardiology Research Center, Cardiovascular Research Institute, Isfahan University of Medical Sciences, Isfahan, Iran; 4 Department of Community Nutrition, School of Nutritional Sciences and Dietetics, Tehran University of Medical Sciences, Tehran, Iran; 5 Epidemiology and Biostatistics Department, Health School, Isfahan University of Medical Sciences, Isfahan, Iran; 6 Nutrition Department, The Ministry of Health and Medical Education, Tehran, Iran; 7 School of Population and Public Health, Faculty of Medicine, University of British Columbia, Vancouver, Canada

**Keywords:** Salt intake, Dietary sodium, Reliability, Validity, Food frequency questionnaire

## Abstract

**Background:**

Few semi-quantitative food frequency questionnaires (SFFQ)s has yet been developed to assess sodium intake in Middle East region. This study was performed to validate a SFFQ for assessment of sodium consumption and food groups΄ contribution to sodium intake.

**Methods:**

This study was performed on 219 healthy participants including 113 adults aged ≥19 years and 106 children aged 6–18 years in Isfahan, Iran. They were administered two SFFQ at the beginning and after 1 year to evaluate the reproducibility. The validity of SFFQ for assessment of sodium intake was compared with 24-h urine sodium and twelve 24-h dietary recalls which were completed monthly during a year as two standard methods.

**Results:**

Correlation coefficient between the contribution of food groups to sodium intake based on SFFQ and 24-h dietary recalls varied from 0.04 for legumes (*P* = 0.667) to 0.47 for added salt (*P* < 0.001). There was a significant correlation between the estimated total sodium intake based on SFFQ and both standard methods (*P* < 0.01). Intraclass correlation coefficient (95% CI) between first and second SFFQ had a diverse range from 0.10 (-0.05, 0.17) for fats and oils to 0.49 (0.28, 0.69) for bread. According to the Bland-Altman plots, we observed an acceptable level of agreement between the two methods for sodium intake.

**Conclusions:**

The SFFQ was a relatively valid and reproducible method for estimating sodium intake. Combination of this SFFQ with a valid prediction of 24-h urinary sodium excretion can be useful in achieving more accurate results.

**Supplementary Information:**

The online version contains supplementary material available at 10.1186/s12937-021-00749-7.

## Introduction

According to United Nations’ summit in the 2011 [1] and World Health organization (WHO) report, a 30% decrease in salt intake by 2025 is one of the 9 goals target and the most cost-effective strategy for non-communicable diseases prevention in different age and gender groups [2]. National initiatives and recommendations for reducing salt consumption in Iranian population need simple and precise tools for salt intake assessment to monitor the impact of their efforts in national surveillance programs and adherence to dietary guidelines. Awareness of which foods are the major contributors of salt is the other crucial subject that could assist the policy makers to target those foods for salt reduction [3].

There is no national report of salt/ sodium intake in Iran. However, there were three 24-h urinary sodium excretion (24hUNa) studies performed in representative adult population samples in the Isfahan, a city in central part of Iran. Daily salt intake based on 24hUNa, in the years 1998, 2001, 2007 and 2013, were 9.5, 9.7, 9.6 and 10.2 g/d, respectively [4]. The major sources of sodium were different in various populations. Processed food contributes greatly to Na intake in the USA and the UK, salt added during home cooking in China [7] and bread in France [9]. However, added salt, bread and cheese were  major sources of sodium in Iran [8].

There are several methods for assessment of sodium intake, and each method has different strengths and limitations [10]. The 24hUNa is an objective measure considered to be the standard by WHO owing to its precision [11]. However, because of high cost, participant burden and difficulty in urine collection, it is deemed cumbersome for repeated measurement in large-scale epidemiological studies. Dietary assessment methods are the alternative techniques. The semi-quantitative food frequency questionnaire (SFFQ) has been proposed as an accurate tool which employed extensively for various reasons including assessment of food and nutrients intake as well as food contribution in nutrients consumption [12]. It is the easiest and least expensive dietary assessment method with low recall bias for evaluation of usual dietary intake in large- scale studies [13]. Although several studies validated SFFQ for various purposes among Iranian population, few SFFQ has yet been developed and validated to assess Na intake in Iran and Middle East region.

The current study was performed to validate SFFQ for salt consumption assessment compared with the standard method of 24hUNa and 24-h dietary recalls (24DRs). Moreover, we aimed to validate the SFFQ for evaluation of total consumption and major sources of sodium and food groups΄ contribution in sodium intake compared to monthly 24DRs during a year as a dietary standard method.

## Methods

### Design and sampling

This cross-sectional study was conducted among on healthy participants aged≥6 years in 2014–2015 by the Cardiovascular Research Institute and previously described in detail [14]. The sample size was calculated 100 in each age group, to obtain correlation coefficient of 0.3 and power of 90%. Healthy individuals ≥6 years of age from Isfahan city in central part of Iran referred to Isfahan Cardiovascular Research Institute (ICRI) clinics for routine check-up were recruited into this study. A total of 366 participants including 168 children aged 6–18 years and 198 adults aged ≥19 years were eligible and scheduled for an initial visit. They provided at least one complete SFFQ, only adults had one 24-h urine collection sample. A complete urine sample was identified by a total 24-h urinary volume 500 mL and no menstruation during the collection period. Inclusion criteria was age ≥ 6 years. All participants with less than nine 24DRs and adults who did not provide 24-h urine sample were excluded. The other exclusion criteria history of diabetes insipidus, special dietary regimen or fasting at the day and time of sampling, history of using diuretics, history of renal insufficiency, menstruation, oral contraceptives use or pregnancy in women, and excessive sweating during the day of urine collection which occurred in unusual situations, such as cooler weather or without any trigger. Totally 147 subjects refused to complete study including dietary assessment interview or 24-h urine and/ or spot urine samples collection. The study was approved by the Isfahan Cardiovascular Research Institute (a WHO collaborative center) ethics committee. Written informed consent and assent were obtained from adult participants and the parents of children, respectively.

### Data collection

Trained health professionals carried out detailed interviews at study baseline to obtain required information about participants’ socioeconomic, demographic characteristics and smoking status. Physical activity was assessed by means of International Physical Activity Questionnaire [15]. The children’s questionnaires were completed with the help of their mothers and also added an time of exercise session per week which was 1.5 h per week to add activity during school hours. However, more physical activity in the school could give rise to recall bias especially among young children.

#### Anthropometric measurements

At the baseline visit, the trained health professionals measured standing height by wall fixed tape without shoes and recorded it to the nearest 0.5 cm. Body weight was measured by Seca scale (Seca 750; Seca GmbH & Co, Hamburg, Germany) with the subjects wearing light clothes, without shoes and recorded it to the nearest 0.5 kg [16].

#### Blood pressure measurement

Blood pressure (BP) was measured, manually by a trained operator using a mercury sphygmomanometer according to a standard protocol [17], twice each from right and left arms in sitting position after 5 min of rest. The first Korotkoff sound was recorded as the systolic BP and the disappearance of the sounds (V phase) was considered as the diastolic BP. The values of BP used in analysis were the recorded mean level of measured BP in higher arm [17].

#### 24-h urine collection

Adult participants was provided by a large sterile plastic container for collection of a 24-h urine sample. They were asked to collect the 24-h urine sample from 7 am to 7 am the next day. After excluding the first void on the first day, they collected the second fasting voiding of a day to first voiding of next day into the large container. The 24-h urine samples were transferred to the ICRI laboratory, which is standardized with a National Reference Laboratory, in the morning (7–10 a. m). To calculate 24hUNa, Na concentration was multiplied by the volume in liters. Urinary Na, potassium (K), and chloride were measured by emission flame photometry (Caretium Medical Instruments Company), while Creatinine (Cr) was measured using Jaffe method (Technical SMA 12–60) [18]. The subjects with incomplete urine sample were excluded. Our criteria for incompleteness urine were: volume < 500 mL; recorded more than one missed void; collection of ≤20 h; 24-h urine Cr (24hUCr) < 20 mg/dL per kg body weight and < 15 mg/dL per kg body weight in males and females aged < 50 years, respectively and 24hUCr < 10 mg/dL per kg body weight and < 7.5 mg/dL per kg body weight for males and females aged ≥50 years, respectively [19, 20]. To reduce our error in prediction, in those participants that the duration of urine collection was above 20 h and less the 24-h, the volume of the measured 24-h urine sample was adjusted for self-reported collection time by dividing total volume collected to self-reported collection time [21].

### Validity and reproducibility of the SFFQ

**Fig. 1:**
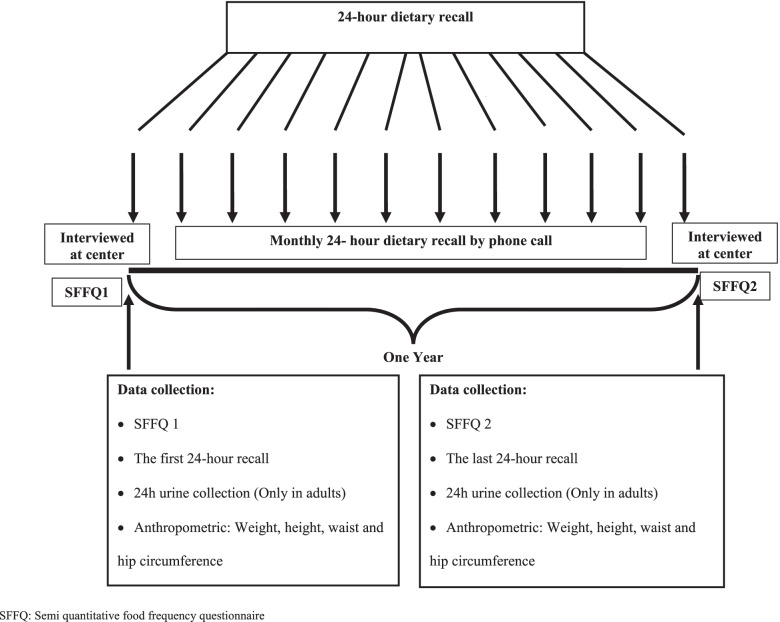
Flowchart of study design and data collection

The SFFQ was completed twice, at the beginning of the study and after 1 year to evaluate the reproducibility. The initial FFQ was accompanied by the first 24DR which was administered by trained dietitian. The validity of SFFQ as a subjective method for assessment of sodium intake compared with the gold standard 24hUNa was carried out at baseline and after 1 year at the end of the study (Fig. 1). We utilized twelve 24DRs which were completed monthly during a year as another gold standard for estimation of SFFQ validation for the assessment of total consumption and food groups΄ contribution in sodium intake. Details were presented in previous publication [14]. The first 24DR was completed in person during their baseline visit to ICRI and the rest of the monthly 24DR were completed via phone calls. In children subjects, all nutrition questionnaires were completed from their mothers.

Initially face and content validity of the questionnaire were confirmed by an expert panel consisting of 10 nutrition experts and calculating content validity ratio (CVR) and content validity index (CVI) [14]. The final SFFQ contained 165 food items were categorized into 16 groups based on sodium content including: 1) dairy (except cheese), 2) cheese, 3) fruits, 4) pickles, 5) vegetables, 6) bread, 7) other cereals (rice, pasta and potato), 8) Meat, 9) legumes, 10) canned foods, 11) fast food, 12) nuts and seeds, 13) sweets and soft drinks, 14) junk foods, 15) oils and fats and 16) sauces. Respondents were asked how many times they consume the foods, scoring the frequency in ten option categories (‘seldom/never’, ‘1 per month’, ‘2–3 per month’, ‘1 per week’, ‘2–3 per week’, ‘4–6 per week’, ‘1 per day’, ‘2–3 per day’, ‘4–5 per day’ and ‘6 or more per day’). All reported numbers were converted to daily frequency and multiplied by the serving number and gram weight of each serving. Seldom and never were calculated as “zero”.

#### Dietary intake analyses

The 24DRS and SFFQ were coded by giving a gram weight to every portion reported. Using Iranian Food Consumption Program (IFCP) designed by ICRI [22], the nutrient and food group intakes were calculated. It has a research quality nutrient database analyzing nutrients and calories for a variety of food items using the Iranian food composition table [23], which was translated to Persian and modified based on USDA National Nutrient Database. Sodium content of some Iranian food in food composition table were modified based on food analysis which had been measured in previous study including different kind of cheese, sausage, bread, biscuit, salty snack, salty nut and seed [24, 25]. In the case of unavailable nutrient values of cooked food in Iranian food composition table, using conversion factors, we calculated nutrient values based on those of raw foods in the processing of both 24DRs and SFFQ. Trained nutritionists assisted in fulfillment and rechecking to complete missing data, checking unclear and unlikely reports within a week with participants and then data entry of the assembled dietary questionnaire. The reproducibility of the questionnaire was obtained through a test and re-test process and completing the SFFQ twice at the beginning and after 1 year of the study.

### Statistical analysis

We calculated the means and SDs for estimated sodium intake from SFFQ as examined method, 24DR and measured 24hUNa as standard methods. To assess agreement between SFFQ and standard methods, paired t-test with logarithmic transformations were conducted to examine the difference in estimated sodium intake based on SFFQ and standard methods including 24hUNa and 24DRs. Using raw data, Spearman’s test and intraclass correlation (ICC) were utilized to evaluate the correlation between sodium intake assessed by SFFQ and standard methods. The Bland-Altman analysis of agreement method was used to estimate the mean bias and 95% limits of agreement between SFFQ and standard methods. For this method, the difference of estimated sodium from SFFQ minus 24DRs or 24hUNa for each participant is plotted against the mean of the two methods. Cross classification between SFFQ and both standard methods were evaluated by calculating frequency of participants in the same adjacent and opposite categories of SFFQ and each standard method. All statistical analyses were performed with SPSS for Windows 19.0 (SPSS Inc., Chicago, IL, USA). *P* values less than 0.05 were considered statistically significant.

## Results

After exclusion of subjects with incomplete dietary recalls and urine samples, a total of 113 adults (60 women and 53 men) and 106 children (50 girls and 56 boys) were included in the final analysis. Table 1 illustrates the subjects’ socio-demographic characteristics. The mean age of adults was 39.93 ± 10.77 while in children was 12.35 ± 3.21 years. Generally, 46.9% of adults were composed of male participants and 52.8% of children were boys.

**Table 1:** Basic characteristics of participants based on gender in adults, children and adolescents

	Adults ***n*** = 113	Children ***n*** = 106
**Age (year)**	39.93 ± 10.77	12.35 ± 3.21
**Sex (Male) n (%)**	53 (46.9)	56 (52.8)
**Marital status n (%)**		
Single	18 (15.9)	99 (93.4)
Married	91 (80.5)	1 (6.6)
Spouse (dead/ divorced)	4 (3.6)	–
**Education n (%)**		
Illiterate and elementary school	15 (13.2)	–
Guidance school	22(19.4)	–
High school and diploma	39 (34.5)	–
University	37 (32.7)	–
**Education (Father) n (%)**		
Illiterate and elementary school	–	20 (18.8)
Guidance school	–	30 (28.3)
High school and diploma	–	47 (44.3)
University	–	9 (8)
**Education (Mather) n (%)**		
Illiterate and elementary school	–	17 (16)
Guidance school	–	35 (33)
High school and diploma	–	39 (36.8)
University	–	15 (14.2)
**Weight (kg)**	71.82 ± 15.18	44.77 ± 17.31
**Height (cm)**	165.37 ± 10.17	148.43 ± 16.78
**BMI**^**a**^**(kg/m**^**2**^**)**	26.43 ± 4.32	19.58 ± 4.56
**WC**^**b**^**(cm)**	91.36 ± 11.60	70.44 ± 12.37
**Physical activity (METS min/week)**	547.12 ± 216.56	653.42 ± 249.53
**Systolic blood pressure (mmHg)**	115.88 ± 11.00	103.02 ± 12.05
**Diastolic blood pressure (mmHg)**	72.31 ± 8.99	59.54 ± 7.17
**24 – hour urinary Sodium (mg/d)**	4130.8 ± 1741.56	–
**24 – hour urinary Potassium (mg/d)**	3801.72 ± 861.51	–
**24 – hour urinary Creatinine (mg/d)**	1287.85 ± 577.22	–
**24 – hour urinary Volume (ml/d)**	1174.82 ± 671.54	–

^a^*BMI* Body mass index

^b^*WC* Waist circumference

As presented in (Table 2), in both times, the mean intakes of sodium and salt in adults and also children were significantly higher when assessed using SFFQ compared to those from mean 24DRs (all *p* values < 0.01). Also, in the first time, there was no significant difference between the mean of sodium and salt derived from SFFQ when compared to the standard method of 24-h urine samples however, it was significantly higher in the second SFFQ (all *p* values < 0.001). Table 3 shows the Spearman correlation coefficient between the contribution of food groups to sodium intake based on data from first and second SFFQ, with 24DRs in adult population. This correlation varied from non-significant for legumes (0.04) in the first time (*P* = 0.667) to significant correlation for added salt (0.47) in the second time (*P* < 0.001). It was ranges, from non-significant correlation for legumes (0.09, *P* = 0.452) in the first time up to significant correlation for bread in the second time (0.45, *P* < 0.001) among children. There was a significant correlation between the estimated sodium intake based on first and second SFFQ with 24DRs and 24hUNa in adults (*P* < 0.01).

**Table 2:** Mean dietary salt and sodium intake estimated by food frequency questionnaire, 24-h dietary recalls and measured 24-h urinary sodium excretion

	Adults							Children			
	FFQ	24DRs	P^**^	Overestimation (%)	24hUNa	***P***	Overestimation (%)	FFQ	24DRs	***P***	Overestimation (%)
	Mean ± SD^**a**^	Mean ± SD			Mean ± SD			Mean ± SD	Mean ± SD		
**Time 1:**											
**Salt (r/d)**	10.21 ± 1.51	9.13 ± 1.43	0.009	11.7	9.60 ± 1.45	0.081	6.3	9.30 ± 1.34	9.16 ± 1.34	0.002	1.5
**Sodium (mg/d)**	4080.42 ± 602.34	3652.12 ± 578.65	0.008	11.7	3840.12 ± 580.56	0.084	6.3	3720.81 ± 1560.19	3662.32 ± 1559.87	< 0.001	1.5
**Time 2:**											
**Salt (g/d)**	10.32 ± 1.35	9.13 ± 1.43	0.003	13.00	9.12 ± 1.64	< 0.001	13.1	9.29 ± 1.33	9.16 ± 1.34	0.008	1.4
**Sodium (mg/d)**	4128.45 ± 539.17	3652.12 ± 578.65	0.002	13.00	3647.29 ± 654.73	< 0.001	13.1	3716.50 ± 1320.37	3662.82 ± 1559.87	0.001	1.4

*FFQ* Food frequency questionnaire, *24DRs* 24-h dietary recalls, *24hUNa* 24-h urinary sodium excretion

**P P. value

^a^*SD* Standard deviation

**Table 3:** Spearman correlation coefficient between sodium and salt intake estimated by food frequency questionnaires and 24-h dietary recalls based age group

Food groups (g/ d)	Adults				Children			
	Time 1		Time 2		Time 1		Time 2	
	ρ (***P***)^*^	Deattenuated	ρ (***P***)	Deattenuated	ρ (***P***)	Deattenuated	ρ (***P***)	Deattenuated
**Dairy (except cheese)**	0.19 (0.126)	0.24	0.26 (0.011)	0.32	0.24 (0.030)	0.29	0.29 (0.006)	0.33
**Cheese**	0.41 (< 0.001)	0.47	0.45 (< 0.001)	0.52	0.32 (0.003)	0.38	0.42 (< 0.001)	0.48
**Fruits**	0.27 (0.004)	0.32	0.31 (0.002)	0.35	0.11 (0.172)	0.15	0.20 (0.051)	0.25
**Pickles**	0.25 (0.011)	0.33	0.33 (0.002)	0.38	0.27 (0.009)	0.32	0.32 (0.003)	0.36
**Vegetables**	0.07 (0.594)	0.11	0.28 (0.004)	0.33	0.31 (0.004)	0.37	0.36 (0.002)	0.41
**Bread**	0.21 (0.029)	0.30	0.45 (< 0.001)	0.52	0.27 (0.004)	0.34	0.45 (< 0.001)	0.51
**Other cereals**	0.24 (0.045)	0.29	0.29 (0.008)	0.35	0.22 (0.034)	0.28	0.25 (0.026)	0.30
**Meat**	0.23 (0.015)	0.30	0.37 (< 0.001)	0.42	0.23 (0.035)	0.29	0.35 (0.003)	0.39
**Legumes**	0.04 (0.667)	0.10	0.14 (0.192)	0.29	0.09 (0.452)	0.13	0.11 (0.326)	0.15
**Canned foods**	0.21 (0.031)	0.27	0.31 (0.002)	0.39	0.39 (< 0.001)	0.44	0.43 (< 0.001)	0.53
**Fast food**	0.30 (0.004)	0.37	0.42 (< 0.001)	0.49	0.28 (0.014)	0.33	0.44 (< 0.001)	0.49
**Nuts and seeds**	0.27 (0.007)	0.31	0.35 (0.001)	0.41	0.30 (0.006)	0.37	0.32 (0.003)	0.36
**Sweets and soft drinks**	0.35 (< 0.001)	0.39	0.37 (< 0.001)	0.42	0.39 (< 0.001)	0.43	0.42 (< 0.001)	0.47
**Junk foods**	0.26 (0.005)	0.31	0.35 (0.002)	0.41	0.41 (< 0.001)	0.48	0.43 (< 0.001)	0.49
**Oils and fats**	0.13 ( 0.197)	0.17	0.29 (0.009)	0.33	0.25 (0.011)	0.30	0.24 (0.031)	0.28
**Sauces**	0.24 (0.010)	0.29	0.36 (0.001)	0.41	0.24 (0.030)	0.28	0.35 (0.004)	0.40
**Added salt**	0.46 (< 0.001)	0.52	0.47 (< 0.001)	0.53	0.35 (0.001)	0.41	0.40 (< 0.001)	0.46
**Total sodium**^**a**^	0.41 (< 0.001)	0.48	0.65 (< 0.001)	0.69	0.37 (0.001)	0.43	0.61 (< 0.001)	0.66
**Total sodium**^**b**^	0.27 (0.004)	0.32	0.35 (0.002)	0.41	–		–	

^*^ρ (*P*) Spearman correlation coefficient (*P*. value)

^a^Based on 24-h dietary recalls

^b^Based on 24-h urine collection

ICC (95% CI) between first and second SFFQ for assessment of sodium intake had a diverse range from 0.11 (− 0.15, 0.37) for legumes up to 0.49 (0.28, 0.69) for bread in adults. These values ranged among children from 0.10 (-0.05, 0.17) for fats and oils to 0.47 (0.17–0.66) for breads (Table 4). Supplementary Table 1 depicts the Kappa agreement and the frequency of participants in the same, adjacent and opposite quartiles of sodium estimates, based on SFFQ compared with a 24DRs and 24hUNa. The proportion of adult participants in the same quartiles varied from 28.3% for 24hUNa in the first time to 63.7% for 24DRs in the second time, while in the same and adjacent quarters ranged from 72.6% for 24hUNa in the first time to 78.7% for 24DRs in the second time. In the opposite quartile, the percentage of participation ranged for 3.5% 24DRs of the second time up to 8.8% for the 24hUNa in the first time.

**Table 4:** Reproducibility of food frequency questionnaire for assessment of sodium dietary intake

Food groups (g/ d)	Adults		Children	
	ICC (95% CI)^**a**^	***P***-value	ICC (95% CI)	***P***-value
**Dairy (except cheese)**	0.36 (0.18, 0.52)	< 0.001	0.43(0.23, 0.59)	< 0.001
**Cheese**	0.45 (0.28, 0.59)	< 0.001	0.28 (0.06, 0.47)	0.007
**Fruits**	0.30 (0.12, 0.48)	0.001	0.18 (0.04, 0.38)	0.053
**Pickles**	0.34 (0.16, 0.52)	< 0.001	0.37 (0.16, 0.55)	< 0.001
**Vegetables**	0.31 (0.12, 0.49)	0.001	0.39 (0.19, 0.56)	< 0.001
**Bread**	0.49 (0.28, 0.69)	< 0.001	0.47 (0.17, 0.66)	< 0.001
**Other cereals**	0.31 (0.13, 0.50)	< 0.001	0.36 (0.16, 0.54)	< 0.001
**Meat**	0.46 (0.25, 0.68)	< 0.001	0.45 (0.24, 0.65)	< 0.001
**Legumes**	0.11 (−0.15, 0.37)	0.179	0.17 (−0.04, –0.38)	0.061
**Canned foods**	0.33 (0.15, 0.52)	< 0.001	0.21 (0.07, 0.35)	0.008
**Fast food**	0.35 (0.18, 0.55)	< 0.001	0.24 (0.05, 0.45)	0.005
**Nuts and seeds**	0.21 (0.01, 0.40)	0.032	0.20 (0.03, 0.42)	0.035
**Sweets and soft drinks**	0.32 (0.13, 0.49)	< 0.001	0.43 (0.29, 0.63)	< 0.001
**Junk foods**	0.36 (0.17, 0.54)	< 0.001	0.40 (0.21, 0.57)	< 0.001
**Fats and oils**	0.19 (0.10, 0.18)	0.166	0.10 (− 0.05, 0.27)	0.058
**Sauces**	0.20 (0.005, 0.37)	0.031	0.18 (0.03, 0.36)	0.037
**Added salt**	0.48 (0.28, 0.71)	< 0.001	0.34 (0.22– 0.58)	< 0.001
**Total sodium**	0.43 (0.24, 0.63)	< 0.001	0.35 (0.17, 0.43)	0.001

^a^*ICC (95%)* Intraclass correlation coefficient (95% confidence interval)

**Fig. 2:**
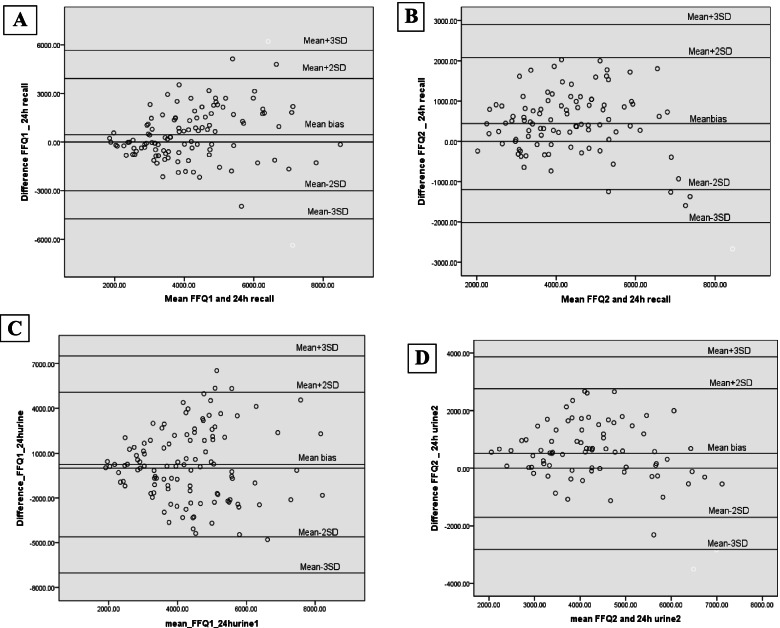
Bland-Altman plots of the mean bias (difference in agreement) between sodium intake assessed by food frequency questionnaire and 24-h recall (**A**) Time 1, (**B**) Time 2 or measured 24-h urinary sodium excretion (**C**) Time 1, (**D**) Time 2 in adult participants. The mean bias for each individual is the assessed sodium intake by food frequency questionnaire minus assessed by 24-h recall (**A** and **B**) or minus measured 24-h urinary excretion (**C** and **D**) and is plotted against the mean of sodium intake assessed by food frequency questionnaire and 24-h recall (**A** and **B**) or assessed by food frequency questionnaire and measured 24-h urinary sodium excretion (**C** and **D**)

**Fig. 3:**
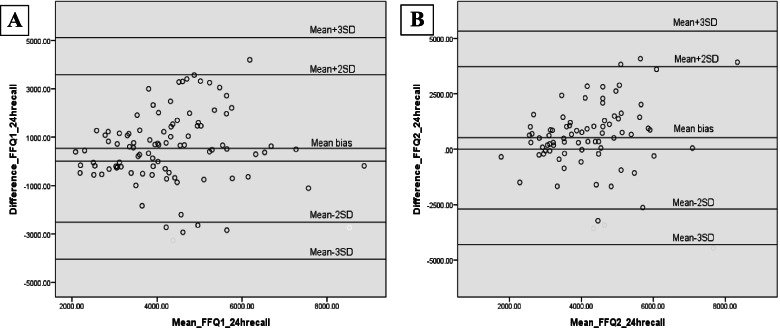
Bland-Altman plots of the mean bias (difference in agreement) between sodium intake assessed by food frequency questionnaire and 24-h recall (**A**) Time 1, (**B**) Time 2 in children and adolescent participants. The mean bias for each individual is the assessed sodium intake by food frequency questionnaire minus assessed by 24-h recall and is plotted against the mean of sodium intake assessed by food frequency questionnaire and 24-h recall

According to the Bland-Altman plots, the mean bias of sodium intake based on SFFQ compared to 24DRs was 453.7 mg/day in adults. Also, the mean bias of sodium intake according to the second SFFQ was 443.8 mg/day in adults when compared to the 24DRs. In adults, the mean bias of sodium estimation based on SFFQ compared to 24hUNa was 239 mg /day and 525.1 mg/day in the first and second times, respectively (Fig. 2). In children, the mean bias of estimated sodium intake was 532.4 mg/day based on first SFFQ and 517.1 mg /day based on second SFFQ compared to the 24DRs (Fig. 3).

## Discussion

The results of this study showed that the SFFQ had a reasonable relative validity in determining the sodium or salt intake in adults, children in comparison with the standard method of 24DRs. The validity of SFFQ was relatively good in adults as compared to the 24hUNa. In this study, SFFQ overestimated the sodium intake compared to the other two standard methods. The overestimation of nutrients by SFFQ compared to other dietary assessments has been previously reported by several studies [27–29]. Reproducibility of the SFFQ for assessment of sodium intake was acceptable. Although the validity of SFFQ was good but according to the Bland-Altman plots, the current bias between SFFQ and standard methods was about 500 mg sodium which was equivalent to 1.2 g of salt. Since the mean of salt intake per day was higher than 10 g [4], we speculate 1 g of salt intake bias is not high. we used the Bland-Altman method which ascertained good agreement between SFFQ and 24DRs. The Bland-Altman method was applied to discover the bias and limits of agreement (LOA) for measures estimated by two methods. Although the mean and interval estimate for bias suggested an over-estimation for sodium intakes in both adults and children, we observed that less than 5%of people fell outside the LOA, which shows good agreement between examined and references methods [31, 32].

Various studies indicated a wide range of validity varying from 0.04 to 0.91 for dietary assessment methods compared to 24hUNa [33, 34, 35]. According to a pooled analysis of Freedman et al. of five validation studies, the mean validity of SFFQ for estimation sodium intake was 0.16 and the underestimation of the SFFQ compared to 24hUNa was 5.6% [36]. The current study found a similar cross-classification agreement and higher validity and of SFFQ for sodium assessment than Xu et al’ s study among Chinese women [35], while relatively less reproducibility. This is probably because of the short interval of two-week between the two SFFQs in Xu et al’ s study [35]. Sasaki et al. [33] showed a less validity of SFFQ for assessment of sodium intake compared to 24hUNa than the current study. Consistent to our findings, Reinivuo et al. [38] reported that the classification of individuals in the same and adjacent quartiles was relatively comparable in both methods. Similarly, several studies conveyed overestimation of sodium intake by dietary assessment compared to the 24hUNa [3, 38, 39]. It could be reasonable since about 90% of total sodium intake might be excreted from urine [39, 40]. On the contrary, the underestimation of SFFQ observed in the Finnish study [37] might have been due to collecting urine samples on Sundays, however most recall days were weekdays. It was reflecting a more consumption of high content of sodium foods during the weekends. In the current study, we estimated discretionary salt which was added at table and cooking through questioning about the weight of salt packages, the number of households, and the period of time that each salt package is used [41, 42]. In the line with previous studies, adding these questions to SFFQ improved the validity and reproducibility of the questionnaire [41, 42]. Our SFFQ was also valid in estimating the contribution of major food sources in sodium intake including added salt, bread, cheese meet, fast food, canned food, nuts and seeds, sweet and soft drinks and junk food, sauce and salty vegetables due to reasonable correlation coefficients between the SFFQ and the standard method of 24DRs in both age groups.

 A study among Belgian school-aged children reported that the validity of SFFQ for assessment of food intake compared to a 24DRs varied from 0.10 for potato to 0.65 for skimmed milk [44]. Similar to our study, they indicated an overestimation of foods such as cereals, beverages, and dairy products [44]. Fumagalli et al. similarly reported that the validity of a SFFQ assessing against 24DRs in children (5–10 yrs) ranged between 0.5–0.7 for most nutrients, however it was low and prone to overestimation of sodium [45].

Possible causes of the inconsistency can be attributed to the daily changes in 24-h urine samples, individuals’ recalling errors during dietary assessments, and lack of completeness and accuracy of food composition tables. Recently Titze et al. suggested that salt can be stored gradually and in a great extent in the inner layer of skin. Therefore, the homeostasis of intercellular sodium cannot be confined to the kidneys, and thus, the sodium estimates by 24-h urine collection might not be accurate [46]. Further potential explanations for these dissimilarities can be due to the excretion of sodium via sweat, which varies greatly depending on the type of weather and physical activity [38]. Also, underestimations in 24-urine collection can simply occur through errors in urine collection methods or the loss of urine volumes. These errors were avoided in the current and previous studies [46] by calculating 24-h creatinine/weight ratio, and questioning about the individuals’ complete urine collection. On the other hand, there are several errors related to the SFFQ such as, lack of completeness and accuracy of food composition tables, errors in individuals’ reports, different sodium content in food items, and daily alterations in diet [32]. Precise measurement of sodium intake is rather challenging, due to diverse distribution of sodium in foods, widespread use of sodium compounds in processed foods as well as drinking water, and high consumption of salt at the table [42]. The current study showed a wide range of reproducibility for assessment of sodium intake in various food groups. It may be caused by more difficulty in recalling intake of food groups like legume, nut and seed, fat and oil and sauce, especially that most of these food groups have low sodium content. Hence, a little recall bias could result of low ICC for reproducibility.

We used the latest food composition table with primary sources being the closest to Iranian food and cuisine. The table was enhanced by adding the sodium content of sodium-containing foods, measured in previous studies [24, 25]. Over- and underestimations in sodium intake has been similarly reported by various studies, however since the most national community-based studies examined the trend of salt intake and also have categorized the people based on nutrients such as sodium, the error in the amounts of intake, when it does not correlate with the high and low levels of intake, is negligible.

### Strengths and limitations

The first strength was encompassing two standard methods including 24DRs and 24hUNa. The second strength was the wide range of age group of study population 6 years and over. This study has also accounted for the seasonal variation, hence collecting twelve 24DRS, as a dietary standard method, during 1 year. The limitations of this study included recall bias of in SFFQ and 24DRs, overestimation of SFFQ and using single 24-h urine collection. Due to examining physical activity of children by asking their mother, another recalll bias was done in assessing physical activity of children in the school. Finally, a larger study population could have been a valuable asset to increase the capability and accuracy the findings.

## Conclusion

The present study suggested that SFFQ was a relatively valid and reproducible method for estimating total sodium intake and major sources of its intake in both adults and children. However, this method overestimated the sodium intake. Since each method has its own strengths and weaknesses in assessments of food consumption, a combination of two or more methods will be useful in achieving more accurate results. Hence, the use of a valid SFFQ along with predicting 24UNa using a spot urine collection and valid formula can be effective in accurate estimation of sodium intake.

## Supplementary Information

**Additional file 1: Supplementary Table 1.** Kappa agreement and Cross classification of estimated sodium intakes from food frequency questionnaire versus 24-h dietary recalls and 24-h urine collection.

## Data Availability

The authors confirm that the data supporting the findings of this study are available.

## Abbreviations

WHO: World Health Organization
24hUNa: 24-h urinary sodium excretion
SFFQ: Semi-quantitative food frequency questionnaire
24DRs: 24-h dietary recalls
ICRI: Isfahan Cardiovascular Research Institute
BP: Blood pressure
CVR: Content validity ratio
CVI: Content validity index
IFCP: Iranian Food Consumption Program
ICC: Intraclass correlation
