# Supplementary material for: Validation and reproducibility of a semi-qualitative food frequency questionnaire for assessment of sodium intake in Iranian population
Source: Nutr J. 2022 Feb 4;21:9. doi: 10.1186/s12937-021-00749-7 (PMC8815124; doi:10.1186/s12937-021-00749-7)
Supplement: Supplementary file 1 — Additional file 1: Supplementary Table 1. Kappa agreement and Cross classification of estimated sodium intakes from food frequency questionnaire versus 24-h dietary recalls and 24-h urine collection. [file 12937_2021_749_MOESM1_ESM.docx]

**Supplementary Table 1: Kappa agreement and Cross classification of estimated dietary intakes between 24-hour dietary recalls and food frequency questionnaire**

|  | **Adults** | | | | **Children** | | | |
| --- | --- | --- | --- | --- | --- | --- | --- | --- |
|  | **Kappa agreement** | **Cross-classification** | | | **Kappa agreement** | **Cross-classification** | | |
|  |  | **Same Quartile**  **n (%)** | **Same/ Adjacent Quartile**  **n (%)** | **Opposite Quartile**  **n (%)** |  | **Same Quartile**  **n (%)** | **Same/ Adjacent Quartile**  **n (%)** | **Opposite Quartile**  **n (%)** |
| **Time 1:** |  |  |  |  |  |  |  |  |
| **24DRs** | 0.34 (<0.001) | 71 (62.8) | 36 (31.9) | 6 (5.3) | 0.22 (0.21) | 47 (44.3) | 54 (50.9) | 5 (4.7) |
| **24hUNa** | 0.20 (0.027) | 32 (28.3) | 71 (62.8) | 10 (8.8) | - | - | - | - |
| **Time 2:** |  |  |  |  |  |  |  |  |
| **24DRs** | 0.45 (<0.001) | 72 (63.7) | 37 (32.7) | 4 (3.5) | 0.33 (0.001) | 53 (50) | 50 (47.2) | 3 (2.8) |
| **24hUNa** | 0.28 (0.009) | 39 (34.5) | 83 (58.4) | 8 (7.1) |  |  |  |  |

FFQ: Food frequency questionnaire; 24DRs: 24-hour dietary recalls; 24hUNa: 24-hour urinary sodium excretion
